# A chemical, crystallographic and magnetic characterisation of individual iron-oxide grains in Hawaiian lavas

**DOI:** 10.1038/sdata.2018.162

**Published:** 2018-08-14

**Authors:** Geertje W. ter Maat, Gillian M. Pennock, Lennart V. de Groot

**Affiliations:** 1 Paleomagnetic laboratory Fort Hoofddijk, department of Earth Sciences, Utrecht University, Budapestlaan 17, 3584 CD Utrecht, the Netherlands; 2 Department of Geoscience and Petroleum, NTNU, 7491 Trondheim, Norway; 3 Structural geology, department of Earth Sciences, Utrecht University, Budapestlaan 4, 3584 CD Utrecht, the Netherlands

**Keywords:** Mineralogy, Palaeomagnetism

## Abstract

Our knowledge on the behaviour of the geomagnetic field through time critically depends on how information of the past state of the field is recorded by, and stored in iron-bearing minerals such as magnetite. For small, single domain grains these processes are described by classical Néel theory, but the magnetic behaviour of larger, pseudo-single domain or multidomain grains, still is enigmatic. Here we present a chemical, crystallographic and magnetic characterisation of three to six individual, large (~3–10 μm) iron-oxide grains from eleven different flows sampled on the Big Island of Hawai’i. These grains were all subjected to a Magnetic Force Microscopy study to characterise their magnetic domain structure; a Microprobe analyses to assess their chemical composition; and a Scanning Electron Microscopy study to identify phases and crystallographic orientations. This comprehensive dataset enables systematic analyses of their magnetic behaviour as function of chemistry and forms the basis for future micromagnetic modelling studies eventually contributing to the development of a fundamental theory of magnetic behaviour in large iron-oxide grains.

## Background & Summary

Igneous rocks are the only source of information on the past state of the Earth’s magnetic field that are available around the globe and throughout geological history. As lava solidifies to form volcanic products such as basalt or volcanic glass, the iron-bearing minerals cool through their Curie temperature, and hence become magnetized due to the ambient (Earth’s) magnetic field at that time. Volcanic edifices with a high eruption frequency are therefore archives of regional variations of the geomagnetic field. The palaeodirection of the field is generally straightforward to obtain by averaging the direction of the magnetic vector stored in a number of independently oriented samples from a single cooling unit. Deriving information on the intensity of the field, however, is notoriously difficult. The theories of Néel^1,2^ and Thellier^3^ to derive such palaeointensities are *sensu stricto* only applicable to small, single domain magnetic particles. The vast majority of remanence carrying grains in extrusive igneous rocks, however, are much larger, pseudo-single or multidomain, grains. The magnetic processes governing the recording, storage, and preservation of magnetic signals in these larger grains are much more complex and still largely enigmatic. Nevertheless, their complex micro-magnetic behaviour is known to hamper palaeointensity experiments, and lowers the success rate of such experiments often to <20% (ref. 4). Progressing our knowledge on the physical processes inside pseudo-single domain and multidomain grains is paramount to improve this success rate and fully unlock the palaeomagnetic information stored in the archives formed by well-dated cooling units in volcanic edifices.

Although research into understanding rock-magnetic processes in large magnetic grains has been going on since the 1980s (refs 5,6), advances in analytical techniques and laboratory equipment recently allowed for a steady increase in the pace at which our knowledge progresses. Current efforts are twofold: first, high-end techniques such as Magnetic Force Microscopy^7–11^ and Electron Holography^12–15^ are used to visualize, map and trace changes in magnetic domain state within particles as function of temperature, time and/or applied magnetic field. Second, micro-magnetic computer models are used to reconstruct and/or predict the three dimensional domain structures within such grains^16–18^. Only very recently these two lines of research could be combined^19–22^, i.e. micro-magnetic computer models are now able to resolve and predict the magnetic domain states that are observed in naturally occurring minerals.

The micro-magnetic behaviour of a grain’s magnetic moment not only depends on its size and shape, but also on its chemical, crystallographic and magnetic properties; e.g. the chemical composition determines the grain’s Curie temperature, and the crystallographic orientation dictates the easy axes of the magnetisation. Furthermore, magnetic domain walls may be pinned at imperfections in the crystal lattice or by zones of differing oxidation state (e.g. oxidation lamellae). To model the magnetic domain state of a natural grain, information on all these properties must be available. Here we present a dataset that provides constraints on all the aforementioned properties for four to seven grains from eleven different lava flows from the Big Island of Hawai’i (Table 1). The grains’ diameters are >3 μm, hence represent pseudo-single domain to multidomain grains and were randomly selected. The magnetic moments of these grains are thought to carry a significant portion of the samples’ (natural) remanent magnetization^11,23^. The samples were taken from a collection that was sampled for a palaeointensity study^24^ and therefore represent natural samples as are typically used for palaeomagnetic studies. Samples from these sites were subjected to an extensive rock-magnetic and palaeointensity study before^24^, hence their bulk magnetic behaviour is well known. The dataset containing chemical, crystallographic, and magnetic characterisations for a number of representative grains presented here enables various studies in micro-magnetic modelling, and fundamental rock-magnetism.

**Figure 1:**
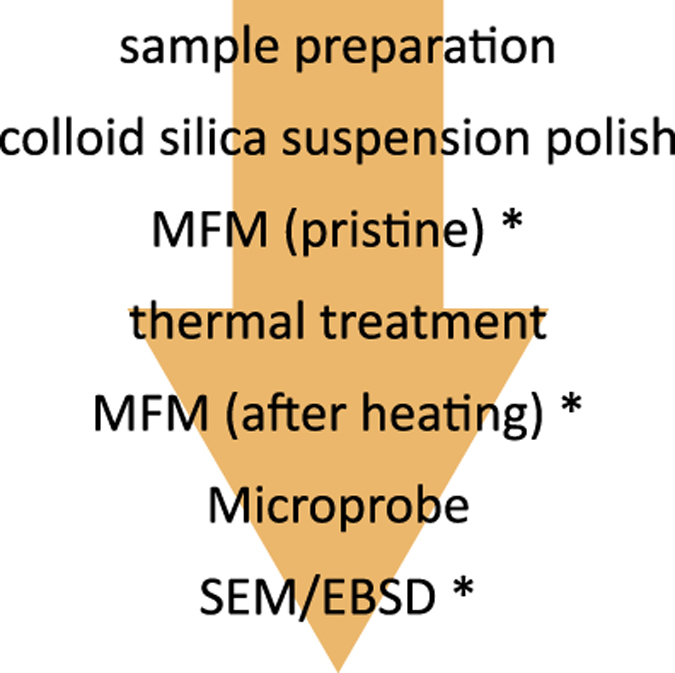
Workflow of the different analyses. Analyses marked with * were only done on a subset of the grains in this study.

The sizes and two-dimensional shapes of the magnetic remanence bearing iron-oxides in the samples were determined by both light and scanning electron (SEM) microscopy. An Electron Backscattered Diffraction (EBSD) study on a high-end SEM yielded information on the crystallographic orientation of the grains and their lattice; a Microprobe was used to determine the chemical contribution of the grains; and the magnetic moment perpendicular to the samples’ surface was imaged with a Magnetic Force Microscope (MFM). The MFM analyses were done both on the pristine samples and after a heating step, to simulate the changes in magnetic domain state that can occur during palaeointensity experiments (Fig. 1).

## Methods

### Sample preparation

For each site in this study two samples were prepared from standard palaeomagnetic drill cores (2.45 cm in diameter, up to 10 cm long): one thin section for light microscopy and one ‘polished surface’ for the SEM, Microprobe and MFM studies. The thin section was used for a first assessment of the typical grain sizes and mineralogy using a microscope; since it was not used for other experiments the thin section will not be discussed here. The polished surface is a disk of 1 cm in diameter and 2 mm thick that was given an extra-fine polish using a colloid silica suspension after the standard polishing to remove any (magnetic) surface strain induced by polishing with traditional pads^11^. The grains of interest were never extracted from the samples, i.e. they are embedded in their original matrix during all experiments in this study. Grains on the surface of the polished surface were cut but the remainder of the grains is intact, i.e. the grains may extend into the sample, and have a volume.

### Light Microscopy

Both the polished surfaces and thin sections were analysed with a microscope, a Leica DM-R with attached digital camera. As the polished surfaces are opaque they were analysed using a reflected light; the thin sections were studied with transmitting light. The petrographic composition, size of phenocrysts and the composition of the groundmass of the thin sections were assessed; hence they were analysed under cross-polarized light (XPL). The microscope study on the polished surfaces was mainly done to confirm the presence of titaniferous iron oxide grains, and analyse their shapes, i.e. discriminate between the occurrence of cubic or spherical grains on one side, and dendritic or cruciform grains on the other. Furthermore, these images were used throughout this study as a map to keep track of the grains selected for the other analyses (SEM, EBSD, Microprobe, and MFM).

### Magnetic Force Microscopy

To visualize the magnetic domains within selected grains in the polished surfaces we used a Nanoscope Multimode AFM/MFM (Veeco Nanoscope IIIa) with hard magnetic point probes (MFM-Reflex coating, tip radius <50 nm, resonance frequency 75 kHZ and force constant 2.8 N/m). The images cover an area of 14.5 by 14.5 μm, with a resolution of 512 by 512 pixels, hence every pixel represents 28.3×28.3 nm. Intrinsic smoothing effects due to the tip radius and lift height determine the effective resolving power of the MFM images. Per sample 4 to 6 grains are imaged. In order to exclude direct magnetic interaction between the grains the grains were selected tens to hundreds of micrometres apart. The MFM was used in interleave mode, a technique that analyses each trace twice: first in tapping mode and second in lift mode. The tapping mode assesses the topography of the grain i.e. yields an Atomic Force Microscopy (AFM) image of the area of interest. The subsequent lift mode is used to measure the stray magnetic field perpendicular to the polished surface with the tip 40 nm above the sample in phase mode. A comparison between the tapping mode and lift mode images identifies potential correlations between magnetic structures and the topography of the sample, e.g. scratches may give rise to local magnetic stray fields from the sample. Subtle surface structures due to changes in composition (e.g. hematite-ilmenite lamellae) can be observed, in addition to the observations in the SEM images. Also structures which may influence the domain structure by domain pinning, e.g. scratches or fractures, are identified. The MFM images of the grains of interest were obtained using a mask derived from the AFM image obtained simultaneously, e.g. the shape of the iron-oxide grain was obtained from the AFM and to cut the magnetic map of the surface of the grain from the entire scan. This removes the expression of stray fields on the edges of the iron-oxide grains that may hamper the interpretation of the MFM images. The full scans are also available in the repository.

It must be emphasized that MFM is a relative technique, changes in e.g. tip age and magnetization influence the interaction between the magnetization of the tip and the sample surface, and hence the colours of the image. The structures (e.g. domain sizes, and shapes) can be compared between images; but colour intensity differences between images should never be evaluated in absolute terms. All images were subjected to a single low-pass filter to reduce high-frequency noise present in some of the images as a result of resonance of the tip.

All samples were subjected to the MFM study in their pristine ‘natural remanent magnetization (NRM)’ state; although it must be noted that cutting and polishing the samples may influence the magnetic state of the surface grains; e.g. surface closure domains may form as a result of changing the shape of the grains. To assess changes in magnetic domain state after a heating step as typical to thermal palaeointensity studies all grains were re-analysed with the MFM after heating them to a set-temperature in the presence of a bias field of the same magnitude as the NRM imparting field (if known)-i.e. giving the samples a partial thermoremanent magnetization (pTRM). The temperatures were chosen per sample based on the palaeointensity experiments and ‘ARM-tests’^25^ performed by de Groot *et al*.^24^ and ranged from 150 °C to 280 °C (Table 1). Before imparting the pTRM, the samples were encapsulated in glass tubes. The glass tubes were flushed with argon and then made vacuum to reduce the risk of oxidation during heating. The NRM direction of the samples was measured using a cryogenic magnetometer (2G DC-SQUID); the samples were then placed in an ASC TD-48SC thermal demagnetizer to be heated. During heating and cooling, a magnetic field equal to the palaeofield the samples cooled in was applied parallel to the NRM directions. The field in which the samples were heated is slightly different for all 11 samples due to age differences of the samples and range from ~36 to ~40 μT (Table 1). After imparting the pTRM the same grains as imaged before the heating step are imaged again by MFM. These images are all made within 24 h after imparting the pTRM to reduce the long-term viscous effects as recently observed by Shaar *et al*.^26^, and visualized by de Groot *et al*.^11^.

### Microprobe

The chemical composition of up to 20 titaniferous iron-oxide grains per sample (including the grains that were studied using MFM and SEM/EBSD) was analysed using a JEOL-8600 Superprobe microprobe. As the Microprobe may damage the surface of the samples while measuring, the Microprobe experiments were done after the MFM experiments. For each grain the element ratio of the spinel-suite (Si, Ti, Al, Fe, Mg, Mn and Cr) was measured in an energy dispersive analysis. Oxygen was not measured directly but calculated on the basis of oxide compositions for the appropriate valence of the metal ion. Based on the ratios of the elements and their valence and the assumption that the grains are unoxidized, the Fe 2+/Fe 3+ ratio can be determined. From this ratio it can be inferred whether or not the particles are partly oxidized (maghemitized)^27^. Before measuring, the polished samples were carbon coated. The accelerating voltage was set to 15 keV, the current 19 nA and the spot size was <1 μm. All grains were selected by hand due to their small size (~3–10 μm). As the thickness of the grains at the spots analysed is unknown, the volume analysed by the Microprobe may contain parts of underlying or neighbouring grains, especially if the spot was close to the edge of the targeted grain. The silica-content in the analyses is a good proxy for whether this occurs: as silica is not expected in the iron-oxides, but is present in almost all other minerals, a high silica content in the analyses indicates that the volume analysed by the microprobe includes parts of surrounding grains as well. Here we use a cut-off of 2.0 atom% Si to consider a Microprobe analysis not representative for the iron-oxide grain and reject it.

### Scanning Electron Microscopy and Electron Backscattered Diffraction

A combination of backscattered electron (BSE) images, orientation contrast (OC) images and electron backscattered diffraction (EBSD) was gathered for nearly all of the grains used for the MFM experiments. The Contrast in BSE images arises from a difference in average atomic number, where a darker BSE region has a lower average atomic number. The OC images show differences in orientations, although the contrast is not quantitative. Very small orienttions differences, less than 0.5°, can already provide contrast. OC imaging is also sensitive to small changes in topography, such as scratches. EBSD measures the orientation of grains.

After completing the MFM and microprobe studies, the crystal orientation of the individual grains were determined using EBSD^28^. All samples were first given a very light polish with colloidal silica for less than a minute to remove the thick carbon coat for microprobe studies that otherwise would have weakened the EBSD signal. This light polish ensured the original surface imaged using MFM was retained for the EBSD study. An FEI XL30 SFEG instrument equipped with an EBSD system (Oxford Instruments HKL Technology, Nordlys detector) was used to generate the signal for the EBSD studies (50 μm final lens aperture, accelerating voltage of 10 kV, beam current of about 2.41 nA (spot size 5), working distance of 15 mm, stage tilt 70°). For most grains the polishing and beam conditions provided good quality EBSD patterns and sufficient conducting carbon remained on the sample for imaging and EBSD pattern collection. Samples were rotated to be in the same orientation as the MFM study. Individual patterns were obtained from fine scale structure typically larger than 0.5 μm in size. A few grains showed a much finer scale structure that gave poor quality patterns. For a few grains, carbon debris obscured the grain preventing collection of an EBSD signal or the remaining carbon coating was insufficient to prevent charging from occurring. EBSD patterns were collected and analysed using Oxford Instruments HKL Technology Channel 5 software. Images of the backscattered (BSE) and orientation contrast (OC)^29^ were collected with three solid state detectors attached to the Nordlys detector, two in the optimum forescatter position and one in the optimum back scatter position. Imaging conditions were the same as used for generating EBSD patterns. In addition, higher resolution BSE images were collected using a solid-state BSE detector attached to the pole piece of the SEM (zero stage tilt, 30 μm final lens aperture, accelerating voltage of 10 kV, beam current of about 1 nA and a working distance of 4 mm).

### Code Availability

No custom codes were used while producing the data presented here.

## Data Records

The data associated with this project is stored in the PANGAEA database (Data Citation 1). Within this dataset, the original (unprocessed) MFM images are available together with the light microscopy images, and the geochemical (i.e. Microprobe) results are provided. These contributions have their own DOIs per site (Table 2). More importantly, we also provide one pdf binder with the results of all analyses comprehensively organized per grain. A sample-specific overview of all analyses done is given in Supplementary File 1.

### Binders

Per site, a pdf-file with the combined results of the analyses is provided (HW#.pdf, with # the site number). These binders are available through either the geochemical, or MFM and microscopy datasets (Table 2), under “Further details”–“Sample description of site HW-#” (with # being the site number). The first page of these binders contains the microscope image with the locations (numbers) of the grains analysed indicated; grains subjected to MFM, SEM/EBSD, and Microprobe analyses are indicated in red, grains only used for Microprobe analyses in yellow. The Microprobe results are given in the table below the microscope image; with the name of the grain in the first column following the convention: HW#-$, in which “#” is the number of the site, and “$” the number of the grain/location. The atom percentages of the following elements are provided: silicon (Si-K), titanium (Ti-K), aluminium (Al-K), iron (Fe-K), magnesium (Mg-K), manganese (Mn-K), chromium (Cr-K), and oxygen (O-K). As silicon is not expected in the iron-oxide grains of interest, excess silicon in the analyses indicates that the beam was not (entirely) on the targeted grain. Here we use 2% silicon as a cut-off to deem a result not entirely representative for the iron-oxide grain; these values are in red.

**Figure 2:**
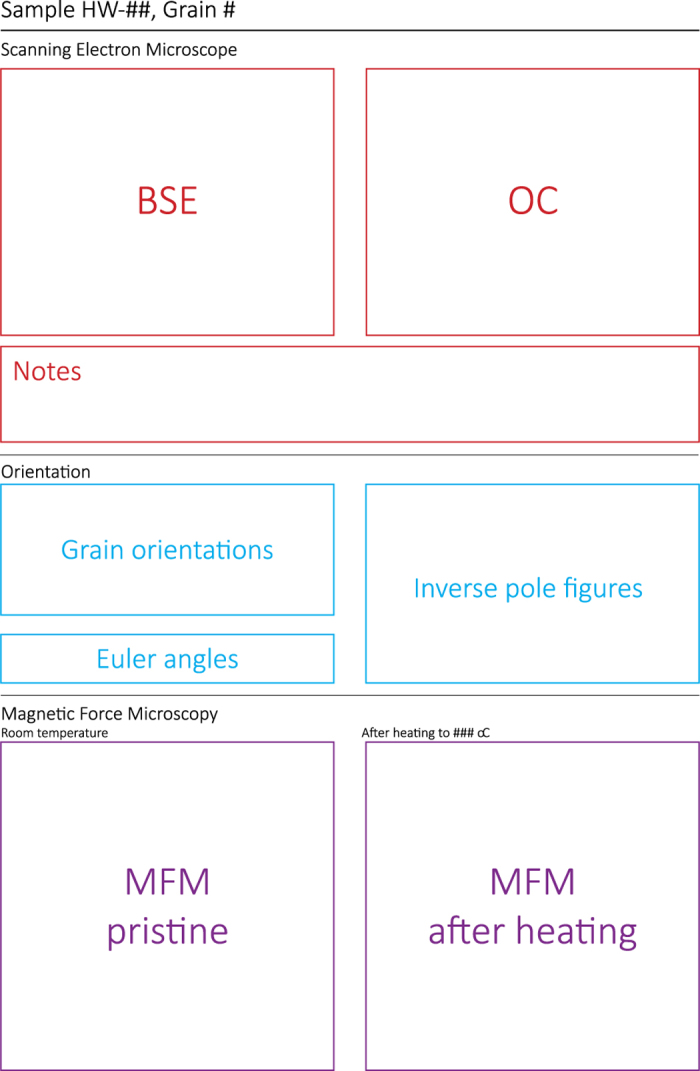
Outline of the overview pages per grain in the data binders. The data are presented in three zones: SEM images and notes are provided in the top part (in red), EBSD information and grain orientations are given in the central part (in blue), and the MFM images are displayed at the bottom of each page (in purple).

The following pages provide the details of the SEM/EBSD, and MFM analyses, with one page per grain (Fig. 2). The pages are split in three parts: the top part (in red in Fig. 2) gives the results of the SEM study, with the Backscattered Electron (BSE) image on the left, and the Orientation Contrast (OC) images on the right. The images are aligned with the EBSD data. Notes arising from the SEM study are below these figures. The locations of the beam used for the EBSD analyses are indicated by numbers in the BSE images. The second part of these pages is used to provide information on the EBSD study (in blue in Fig. 2). Per beam location the orientation of the grain is visualized on the top left, and the Euler angles of this orientation with respect to the detector. The obtained orientations are plotted in Inverse Pole Figures on the right (equal area, upper hemisphere): here the convention for the labels is as follows: “grain number, beam location”, so a label “4,3” would refer to grain 4 of this respective sample, beam location 3 as specified on the BSE image of this particular grain. The bottom part of the pages per grain (in purple, Fig. 2) is used to display the MFM images before (on the left) and after a heating step (on the right). The MFM images are masked using the AFM images and rotated to have the same orientation as in the SEM images, hence the orientation of all the analyses (SEM, EBSD, and MFM) is the same, unless otherwise indicated. The MFM images are displayed on a 1x1 μm grid that is labelled with numbers on the horizontal axis, and with letters on the vertical axis, so it is possible to refer to a particular part of the grain using coordinates such as “C3” or “G8”. The MFM images before and after heating are aligned such that the same part of the grain has the same coordinates in both images, the axis therefore not always start at “1” or “A” in both images. The full, unmasked, MFM images are also available, see below.

### MFM & microscope images

For each site the MFM images and microscopic overview of the grains are provided (Table 2); the file name of the overview is “Optical_HW#.jpg”, with # the site number. The MFM images are named following the convention: “MFM_HW#_grain$_*state*.jpg”, with # the site number, $ the grain number, and ‘*state*’, the magnetic state of the sample, either ‘pristine’ for the NRM state, of ‘afterheating’ for the pTRM state. These MFM images are the original images produced by the Nanoscope, with the AFM (topography) image on the left, and the MFM (magnetic) image on the right. The raw (binary) data of the MFM analyses are also provided under the same DOIs and using the same naming convention as the MFM images, but with extension ‘.001’. The iron-oxides are generally much harder than the surrounding matrix, so they stand out in the AFM image, their outlines where used as a mask to provide the MFM images in the binders. The range of the data scale of the ARM image (from the deepest red to the brightest yellow) is specified following “Z range” below the AFM image; the phase range of the MFM image is specified following “Z range” below the MFM image. Note that MFM is a qualitative analysis and the colour scales should not be compared in absolute terms between different scans.

### Microprobe results

The Microprobe analyses obtained for the sites are available through the respective DOIs (Table 2). The sample names are in the first column, following the convention: HW#-$, in which “#” is the number of the site, and “$” the number of the grain/location, as specified in the microscope images on the first page of the binders per site. The atom percentages of the following elements are provided: silicon (Si), titanium (Ti), aluminium (Al), iron (Fe), magnesium (Mg), manganese (Mn), chromium (Cr), and oxygen (O). Note that oxygen is not measured directly but is calculated based on the oxide compositions for the appropriate valence of the metal ion.

## Technical Validation

### Sample preparation

The volumes of the grains that were analysed in this study were inherently changed during sample preparation. This may lead to a rearrangement of magnetic closure domains on the new surface. Moreover, polishing samples using pads is known to introduce surface strain that influences the domain state on the polished surface^11^. This surface strain can be removed by a last round of polishing using a silica colloid suspension to reveal the ‘natural’ domain state of the sample again (c.f. Supp. Fig. 9 in de Groot *et al*.^11^). All our samples underwent this treatment to supress magnetic domain configurations inferred by polishing-induced surface strain. Hence, the magnetic domain state of the remanence carrying grains in the samples during the initial MFM study represents their NRM state as closely as possible but may have altered by forming closure domains on the surface as a result of sample preparation.

### Magnetic Force Microscopy

The standard sample holder of the Nanoscope Multimode AFM/MFM has an actuator-driven sample stage to aid the approach of the sample to the tip. The sample is kept in place by fixing the sample to a weak-iron disk that sticks to a tiny magnet on top of the actuator. This magnet in combination with the magnetic field of the actuator has the potential to alter the domain state of the samples; hence we opted to use the manual sample stage without the actuator and the magnet to keep the samples in place. With this manual sample stage, however, it is more difficult to keep the sample fully horizontal while approaching the tip, and using this stage increases the danger of destroying the tip by crashing the sample into it. The magnetic field at the location of the MFM tip using the manual sample stage was measured using a Bartington flux gate magnetometer and was found to be similar to the ambient (Earth’s) magnetic field.

The Nanoscope Multimode AFM/MFM is placed on a granite plate that is suspended inside a steel frame to decouple the instrument from ambient noise (i.e. vibration) in the building. The MFM tip was magnetized using the same standard magnet every time while loading a sample in the Nanoscope to ensure a constant magnetization of the tip.

The stability of the magnetic domain structure of the samples was assessed by removing and placing the samples back in the Nanoscope after a 90° rotation. The MFM images before and after handling and rotating the sample did not change, this verifies that the magnetic domain structures that are imaged represent stable, remanent magnetic domain states of the grains.

### Microprobe

The Microprobe was optimized for the ‘spinel task’, a set of elements that is routinely measured on the machine before analysing our samples. The JEOL-8600 Superprobe microprobe does not measure oxygen directly, the oxygen contribution was inferred based on the appropriate valence of the metal ions analysed. During the analyses, the spot size was <1 μm; since the smaller grains in our study are ~3 μm in diameter it is difficult to ensure that the entire chemical characterisation relates to the iron-oxide grain of interest. As small offset in aiming the electron beam, or grains that are too thin, may lead to the inclusion of surrounding minerals in the analysis. Here we used the Si-signal to determine whether only the iron-oxide was targeted, since Si is not expected in the grains of interest, but it is present in almost all other minerals in a basaltic lava. We used a cut-off of 2.0 atom% Si to consider a Microprobe analysis not exclusively representative for the iron-oxide grain.

### Electron Backscattered Diffraction

The obtained phases and orientations of the grains were determined by comparing (i.e. matching) the EBSD patterns to known crystal structures. Titanomagnetite^30^ and ilmenite^31^ were used by the EBSD Channel 5 software to match the EBSD patterns. The first 80 reflectors, ranked in terms of intensity, were sufficient to discriminate between the two phases. For reliable indexing of the phases, a calibration based on patterns from a silicon single crystal, made at the same working distance (15 mm) and accelerating voltage (10 kV) was used. Successful indexing of the EBSD patterns was achieved for most grains based on 8-9 bands in the EBSD pattern. A measure of the reliability of the indexed solution is the mean angular deviation between the calibration simulation and collected EBSD patterns; all indexed solutions were less than 1.3° and were typically between 0.5° to 0.7°. The EBSD phase identification was consistent with the microprobe analyses, and brighter BSE regions were identified as titanomagnetite and darker BSE regions as ilmenite.

## Usage Notes

The samples for this study were selected from a collection gathered on Hawaii in April/May 2010. Limited amounts of pristine sister specimens (taken only centimetres apart from the samples studied here) are available on request, please contact the corresponding author.

## Additional information

**How to cite this article**: Maat, G. W. t. *et al*. A chemical, crystallographic and magnetic characterisation of individual iron-oxide grains in Hawaiian lavas. *Sci. Data* 5:180162 doi: 10.1038/sdata.2018.161 (2018).

**Publisher’s note**: Springer Nature remains neutral with regard to jurisdictional claims in published maps and institutional affiliations.

## Supplementary Material



Supplementary File 1

## Figures and Tables

**Table 1:** Details on the cooling units from which the samples were taken.

**Site /sample**	**Age**	**Latitude (N)**	**Longitude (W)**	**Flow thickness (cm)**	**Depth from top (cm)**	**Temp. (°C)**	Rock-magnetic group*	**ARM-test behaviour**	**Expected palaeointensity (μT)**	**Measured palaeointensities (μT)**
HW-3	1907	19° 4.315’	155° 44.314’	< 270	20	280	M	Overestimate	37.8	
HW-5	1887	19° 4.103’	155° 43.704’	270	190	240	M	Underestimate	38.7	
HW-6	1886	19° 3.558’	155° 41.678’	180	100	280	H	Underestimate	38.7	
HW-7	1886	19° 3.515’	155° 40.874’	80	35	360	H	Correct	38.7	MSP: 38.1
HW-11	1280 ± 70 BP	19° 39.832’	155° 7.113’			150	M	Correct		MSP: 41.9
HW-13C	1960	19° 30.779’	154° 49.127’	108	100	150	M	Correct	36.2	MSP: 31.3
HW-14A	1955	19° 23.938’	154° 55.147’	200	15	240	H	Correct	36.3	IZZI: 40.0
HW-20	1790	19° 26.717’	154° 51.314’	25	15	150	M	Overestimate	41	
HW-22	1843	19° 37.996’	155° 30.527’	50	30	280	H	Overestimate	39.8	
HW-25	670 ± 60 BP	19° 13.767'	155° 27.393'			240	H	Correct		MSP: 44.6
HW-27B	890 ± 60 BP	19° 8.323'	155° 33.113'			240	M	Correct		MSP: 46.8
The site-codes correspond to the codes previously used in de Groot *et al*.^24^; the age is either known from historical sources (until 1843 AD), or based upon radiocarbon dating (pre-1843), for the latter sites the lab age^32,33^ is reported; the location where the samples were taken (latitude, and longitude); the thickness of the flow at the sampling location; the depth from the top of the flow at which the samples were taken; the temperature used for the heating step in the MFM measurements and the ‘ARM-tests’^25^ as described in de Groot *et al*.^24^; the rock-magnetic group^24^; the prediction of the ARM-test^24^; the International Geomagnetic Reference Field (IGRF) intensity for the given age and location for sites until 1843 AD; results of palaeointensity experiments for these sites (‘MSP’: multispecimen-style experiment^34,35^, ‘IZZI’: IZZI-Thellier style experiment^36^) as reported in de Groot *et al*.^24^.										

**Table 2:** DOIs of individual datasets.

**Site/sample**	**Geochemistry**	**MFM & microscope images**
HW-3	10.1594/PANGAEA.886153	10.1594/PANGAEA.886176
HW-5	10.1594/PANGAEA.886160	10.1594/PANGAEA.886177
HW-6	10.1594/PANGAEA.886161	10.1594/PANGAEA.886178
HW-7	10.1594/PANGAEA.886162	10.1594/PANGAEA.886179
HW-11	10.1594/PANGAEA.886154	10.1594/PANGAEA.886167
HW-13C	10.1594/PANGAEA.886155	10.1594/PANGAEA.886168
HW-14A	10.1594/PANGAEA.886156	10.1594/PANGAEA.886169
HW-20	10.1594/PANGAEA.886157	10.1594/PANGAEA.886170
HW-22	10.1594/PANGAEA.886164	10.1594/PANGAEA.886171
HW-25	10.1594/PANGAEA.886158	10.1594/PANGAEA.886172
HW-27B	10.1594/PANGAEA.886159	10.1594/PANGAEA.886175
The geochemical results (i.e. Microprobe data) are provided separately from the MFM and light microscopy images. The binders comprehensively presenting all available data per grain are accessible through either the geochemical, or the MFM and microscope images datasets, under “Further details:”–“Sample description of HW-#”, with # the site number.		
